# The genetic basis of grape and wine aroma

**DOI:** 10.1038/s41438-019-0163-1

**Published:** 2019-07-01

**Authors:** Jerry Lin, Mélanie Massonnet, Dario Cantu

**Affiliations:** Department of Viticulture and Enology, University of California Davis, One Shields Ave, Davis, CA 95616 USA

**Keywords:** Secondary metabolism, Plant genetics

## Abstract

The grape is one of the oldest and most important horticultural crops. Grape and wine aroma has long been of cultural and scientific interest. The diverse compound classes comprising aroma result from multiple biosynthetic pathways. Only fairly recently have researchers begun to elucidate the genetic mechanisms behind the biosynthesis and metabolism of grape volatile compounds. This review summarizes current findings regarding the genetic bases of grape and wine aroma with an aim towards highlighting areas in need of further study. From the literature, we compiled a list of functionally characterized genes involved in berry aroma biosynthesis and present them with their corresponding annotation in the grape reference genome.

## Introduction

With nearly 8 million hectares of vineyards worldwide and a global annual production of around 90 million tons, the grapevine is one of the most important horticultural crops (http://faostat.fao.org). The majority of cultivated grapes belong to *Vitis vinifera* subsp. *vinifera* (hereafter *V. vinifera*); however, cultivars and hybrids of other *Vitis* species, as well as the related genus *Muscadinia* are also grown, particularly in regions where climate and/or disease pressure preclude the cultivation of *V. vinifera*^1–3^. Over its long history of domestication and usage for wine and consumption as fresh and dried fruit, *V. vinifera* has undergone selection for desirable traits such as hermaphroditism, sugar content, berry color, and berry size^4–6^. Attempts to utilize wild non-*vinifera* species, whether as interspecific crosses with *V. vinifera* or as selected cultivars, to overcome environmental and biotic limitations on grape cultivation have been complicated by the presence of negatively perceived flavors, especially in wine production^2,3,7^. On the other hand, there is enormous genetic diversity in cultivated and wild species of grape and with it the potential to generate novel flavor combinations by utilizing that diversity^8–10^.

The aroma, i.e., the olfactory component of flavor, of grapes and wine has been an area of intense interest for many decades. Much has been written regarding topics such as the volatile profiles of different cultivars, the sensory significance of individual compounds, environmental effects on the accumulation of aroma compounds, and the impacts of viticultural and enological treatments on fruit and wine aroma. Advances in analytical techniques together with sensory methodologies have dramatically increased our understanding of the composition and interactions within the complexity that is grape and wine aroma^11^. While variations in aromatic potential between cultivars have long been documented, for many reasons the study of genetic explanations for these differences has lagged behind our knowledge of grape volatile constituents.

Only fairly recently have we been able to unravel the role of specific genes in the origins of grape and wine aroma. Molecular markers have been invaluable in helping researchers understand the inheritance of a number of viticulturally and enologically significant traits including those related to flavor and aroma^10^. Expressed sequence tags (ESTs) have also been instrumental in the identification of genes involved in secondary metabolism^12,13^. More recently, the publication of the Pinot noir and PN40024 genomes has greatly facilitated the identification and characterization of genes involved in many metabolic pathways^14–18^. Ongoing technological advances in sequencing, omics, biotechnology, and phenotyping will further enable researchers to understand the genetic mechanisms behind grape and wine aromas.

The purpose of this review is to summarize the state of our knowledge regarding grapevine genetics in relation to the biosynthesis and modification of volatile compounds. The current review is not intended to be a complete listing of the volatile compounds found in wine but to inform the reader on what has so far been elucidated regarding their genetic basis and to highlight areas that need further study. Although the impact of environmental conditions on the biosynthesis and metabolism of volatile compounds can be considerable, genotype by environment interactions have not yet been thoroughly explored in grape. A number of excellent reviews have been written regarding the diversity of wine and grape aroma compounds, environmental impacts on their biosynthesis, and their chemistry, to which we refer the reader for more information in those areas^11,19–25^. An emphasis has been made here on genes that have been functionally characterized in grapevine. Where little study has been conducted on grapevine, comparable systems in other plants have been provided.

## Grape aroma compounds

The predominate compounds contributing to the aroma profile of grape berries fall into the following categories: mono- and sesquiterpenes, methoxypyrazines, furan derivatives, lipoxygenase pathway products, and phenylpropanoid pathway products. Two classes of compounds, norisoprenoids and volatile sulfur compounds, are important in wine but are generally found as nonvolatile precursors in berries and require further modifications to be perceived^2,3,11,21,26^. During winemaking and subsequent aging processes, volatile compounds and their precursors can undergo enzyme-catalyzed modifications and spontaneous chemical transformations. Further chemical diversity is introduced by wine microorganisms and aging vessels^21^.

This complexity, along with extremely low concentrations of some compounds, significant environmental and temporal effects on volatile concentration and composition, and conjugation of volatile compounds into nonvolatile precursors has made research into the aroma components of grape berries challenging^11,21,27^. The difficulty is compounded in the characterization of genes encoding enzymes that produce aroma compounds or their precursors. In the subsequent sections of this review, we summarize the current knowledge regarding the major groups of grape aroma compounds and genes involved in their biosynthesis. The genes which have been functionally characterized and shown to produce aroma compounds or their precursors are listed in Table 1.

**Table 1:** Genes involved in the biosynthesis and metabolism of grape berry and wine aroma compounds and precursors

Compound class	Gene name^a^	Function	GenBank accession number	Gene ID^b^	References
Monoterpenes	*VviDXS*	1-Deoxy-D-xylulose 5-phosphate synthase	MA161553	VIT_05s0020g02130	Battilana et al.^42^, Duchêne et al.^53^
	*VviTPS52 VviGer*	Geraniol synthase	HM807399	VIT_12s0134g00140	Martin et al.^14^, Matarese et al.^54^
	*VviTPS54 VviLinNer1*	(3*S*)-Linalool/(*E*)-Nerolidol synthase	HM807391	VIT_00s0385g00020	Martin et al.^14^, Matarese et al.^54^
	*VviTPS56 VviLinNer2*	(3*S*)-Linalool/(*E*)-Nerolidol synthase	HM807392	VIT_00s0271g00060	Martin et al.^14^, Zhu et al.^63^
	*VviTPS57 VviLNGl1*	Linalool/(*E*)-Nerolidol/ (*E*,*E*)-Geranyl linalool synthase	HM807394	VIT_00s0372g00040	Martin et al.^14^
	*VviTPS58 VviLNGl3*	Linalool/(*E*)-Nerolidol/ (*E*,*E*)-Geranyl linalool synthase	HM807396	VIT_00s0372g00070	Martin et al.^14^
	*VviTPS61 VviLNGl4*	Linalool/(*E*)-Nerolidol/ (*E*,*E*)-Geranyl linalool synthase	HM807397	VIT_00s0372g00070	Martin et al.^14^
	*VviTPS63 VviLNGl2*	Linalool/(*E*)-Nerolidol/ (*E*,*E*)-Geranyl linalool synthase	HM807395	VIT_00s0847g00020	Martin et al.^14^
	*VviTer*	(-)-α-Terpineol synthase	AAS79351	VIT_13s0067g00370	Martin et al.^12^
	*CYP76F14*	(*E*)-8-Carboxylinalool synthase	CAB85635	VIT_02s0025g04880	Ilc et al.^69^
	*VviGT7*	Glucosyltransferase	XM_002276510.2	VIT_16s0050g01580	Li et al.^58^
	*VviGT14*	Glucosyltransferase	XM_002285734.2	VIT_18s0001g06060	Bönisch et al.^72^, Li et al.^58^
	*VviGT15a*	Glucosyltransferase	XM_002281477.2	VIT_06s0004g05780	Bönisch et al.^72^, Li et al.^58^
	*VviGT15b*	Glucosyltransferase	XM_002281477.2	VIT_06s0004g05780	Bönisch et al.^72^, Li et al.^58^
	*VviGT15c*	Glucosyltransferase	XM_002281477.2	VIT_06s0004g05780	Bönisch et al.^72^, Li et al.^58^
Sesquiterpenes	*VviTPS24 VviGuaS*	Selinene synthase polymorphic variant	XM_002282452	VIT_19s0014g02590	Drew et al.^84^
	*VviSTO2*	Sesquiterpene oxidase α-guaiene 2-oxidase	XM_010646246	VIT_19s0015g00110	Takase et al.^81^
Norisoprenoids	*VviCCD1.1*	Carotene cleavage dioxygenase	KF008001	VIT_13s0064g00810	Lashbrooke et al.^16^, Mathieu et al.^100^
	*VviCCD1.2*	Carotene cleavage dioxygenase	AY856353	VIT_13s0064g00840	Mathieu et al.^100^
	*VviCCD4a*	Carotene cleavage dioxygenase	KF008002	VIT_02s0087g00910	Lashbrooke et al.^16^
	*VVICCD4b*	Carotene cleavage dioxygenase	KF008003	VIT_02s0087g00930	Lashbrooke et al.^16^
Methoxypyrazines	*VviOMT1*	S-Adenosyl-L-Met (SAM)-dependent O-methyltransferase	GQ357167	VIT_12s0059g01790	Dunlevy et al.^135^, Hashizume et al.^134^
	*VviOMT2*	S-Adenosyl-L-Met SAM)-dependent O-methyltransferase	GQ357168	VIT_12s0059g01750	Dunlevy et al.^135^
	*VviOMT3*	S-Adenosyl-L-Met SAM)-dependent O-methyltransferase	KC243500	VIT_03s0038g03090	Dunlevy et al.^133^, Guillaumie et al.^129^
	*VviOMT4*	S-Adenosyl-L-Met SAM)-dependent O-methyltransferase	KC243503	VIT_03s0038g03080	Dunlevy et al.^133^, Guillaumie et al.^129^
Furans	*VviQR* ^c^	Enone reductase	Not available	VIT_01s0127g00740	Sasaki et al.^138^
	*VviGlyT_07 UGT85K14*	Furaneol glucosyltransferase	XM_002268601.2	VIT_00s0324g00050	Sasaki et al.^138^
Volatile thiols	*VviGST3*	Glutathione synthase	EF469244	VIT_12s0028g00920	Kobayashi et al.^157^
	*VviGST4*	Glutathione synthase	AY971515	VIT_04s0079g00690	Kobayashi et al.^157^
	*VviGGT* ^c^	γ-Glutamyltranspeptidase	Not available	VIT_11s0016g02830	Kobayashi et al.^157^
Lipoxygenase	*VviLOXA*	Type II lipoxygenase	XM_002285538 NM_001281094.1	VIT_06s0004g01510	Podolyan et al.^152^
	*VviLOXO*	Type II lipoxygenase	XM_002273222.2	VIT_09s0002g01080	Podolyan et al.^152^
	*VviHPL1*	13-hydroperoxidase	HM627632	VIT_12s0059g01060	Zhu et al.^155^
	*VviHPL2*	9-hydroperoxidase	HM627633	VIT_03s0063g01830	Zhu et al.^155^
	*VviADH1*	Alcohol dehydrogenase	AF194173	VIT_18s0001g15410	Qian et al.^156^, Tesnière et al.^163^
	*VviADH2*	Alcohol dehydrogenase	AF194174	VIT_04s0044g01110	Qian et al.^156^, Tesnière et al.^163^
	*VviADH3*	Alcohol dehydrogenase	AF194175	VIT_18s0001g15450	Tesnière et al.^163^
	*VviAAT*	Alcohol acyltransferase	AAW22989	VIT_09s0018g01490	Qian et al.^156^
Phenylpropanoid	*AMAT*	Anthraniloyl-coenzyme A (CoA): methanol acyltransferase	AY705388	VIT_09s0018g01490	Wang and Luca^8^

*Note*: All genes have been functionally characterized and shown to be expressed in berries during development unless noted

^a^PN40024 alleles are listed for genes for which multiple alleles have been characterized

^b^Best hit in PN40024 V1 annotation

^c^Identified but not functionally characterized

## Terpenoids

Terpenoids represent an extremely diverse class of natural compounds with over 40,000 identified from organisms in every kingdom of life. In plants, multiple processes such as photosynthesis, membrane construction, and growth regulation utilize terpenoids^21,28,29^. The volatile terpenoids are important constituents of plant aromas and play major roles in plant-animal interactions, general defense, and responses to abiotic stresses. In addition, they contribute key components to the fragrance of many flowers, fruit, and herbs^28,30^. Terpenoids consist of multiple C_5_ isoprene units and, despite their diversity, share isopentyl pyrophosphate (IPP) and dimethylallyl pyrophosphate (DMAPP) as precursors^29^. Biosynthesis of terpenoids occurs both in the cytoplasm via the mevalonic acid (MVA) pathway and in the plastid through the methyl-erythritol-phosphate (MEP) pathway^21,28^. Several excellent reviews of both pathways and their elucidation have been written^31–33^. Many of the genes involved in these pathways remain to be identified and functionally characterized in grape^21^. The terpenoids of major importance in grape and wine aroma are the monoterpenes, sesquiterpenes, and indirectly, carotenoids^11,21,22^.

## Monoterpenes

Twenty two different monoterpenes have been identified in grapes and wine; half are linalool derivatives^22^. Monoterpenes are characteristic components of berries during early development but in most cultivars, levels decline below the detection threshold after véraison and are not major contributors to the aroma of ripe berries^11,34,35^. Muscats represent a major exception as their characteristic floral aroma is due to high concentrations of monoterpenes with linalool, geraniol, nerol, α-terpineol, and hotrienol being the most important. Small amounts of other terpenes contribute to varietal differences. Interactions between combinations of monoterpenes and their metabolites result in a spectrum of flavor intensities among Muscat varieties^23,36^.

Grape cultivars can be divided into three general groupings based on total free monoterpene concentration, as well as by monoterpene profile: neutral varieties with very low concentrations such as *V. vinifera* cv. Chardonnay, “aromatic” cultivars with 1–4 mg/L of monoterpenes e.g., *V. vinifera* cv. Riesling, and Muscat types like *V. vinifera* cv. Muscat blanc and cv. Muscat of Alexandria with as much as 6 mg/L of free monoterpenes^37,38^. Profiling 15 monoterpenes is sufficient for discriminating between Riesling and five of its offspring^38^. Overall, monoterpenes are mainly concentrated in the skin with differential distribution depending on the compound. Geraniol and nerol are much more abundant in the skin than in the flesh, while linalool is more generally distributed throughout the fruit^39^. The genetic factors responsible for increasing the accumulation of monoterpenes have long been of interest to grape breeders.

Using simple sequence repeat (SSR) markers, Doligez et al. identified a major quantitative trait locus (QTL) on linkage group (LG) 5 associated with linalool, nerol, and geraniol content within an 8.3 cM interval between markers VRZAG79 and VVC6^40^. Battilana et al. and Duchêne et al. confirmed the location of a QTL on LG 5 associated with total monoterpene levels in two Muscat × neutral mapping populations and two populations of selfed aromatic varieties (*V. vinifera* cv. Muscat Ottonel and cv. Gewürztraminer), respectively^41^. A *V. vinifera* 1-deoxy-D-xylulose 5-phosphate synthase (*VviDXS*) gene was identified as the gene responsible for Muscat character due to its co-localization with the LG 5 QTL associated with monoterpene content^42^. Overall, the *VviDXS*-associated QTL was found to explain 17–93% of the variation in linalool, nerol, and geraniol concentrations^40–42^. This is consistent with the role of *VviDXS*, which as the first enzyme of the MEP pathway catalyzes the formation of the substrates used for monoterpene production.

The *VviDXS* sequence contains a total of 4790 bp in 10 exons and 9 introns and encodes a protein with 716 amino acids^43^. *DXS* is highly conserved in plants and bacteria due to its importance in the MEP pathway^44,45^. *VviDXS* expression in Muscat blanc increases at véraison and peaks at 12 weeks post flowering. Expression of the gene is significantly correlated with the accumulation of free and bound monoterpenes, and both *VviDXS* transcript levels and free monoterpene concentrations decrease late in fruit ripening while bound monoterpenes continue to increase^46^.

Analysis of the *VviDXS* sequence among 148 Muscat, Muscat-like aromatic, and neutral cultivars identified 95 Single Nucleotide Polymorphisms (SNPs), four of which were predicted to affect protein function^43^. A G/T SNP results in replacement of the lysine (K) at position 284 with asparagine (N). Ninety five percent of the Muscat cultivars examined by Emanuelli et al. possessed the K284N mutation with 68 of the 69 Muscat cultivars having the allele in a heterozygous state. The highly aromatic Gewürztraminer and its offspring *V. vinifera* cv. Siegerrebe share a C/T SNP resulting in replacement of arginine with cysteine in one allele of *VviDXS* (R306C). Two aromatic “musqué” clones of otherwise neutral varieties were also found to have non-neutral mutations within *VviDXS*: *V. vinifera* cv. Chasselas musqué has an A/G SNP causing a deletion of amino acid residues 285–289 while a musqué clone of Chardonnay has a T/C SNP replacing the serine at position 272 with proline. The four mutations are located in a region corresponding to the active site of DXS in *Deinococcus radiodurans*^43^. Of the identified mutant *VviDXS* alleles, the Muscat N284 allele has received the most attention from researchers.

To determine the effect of the N284 mutation on *VviDXS* function, Battilana et al. expressed both the N284 and K284 *VviDXS* variants in *E. coli*^46^. Recombinant VviDXS N284 exhibited twice the catalytic efficiency compared with VviDXS K284 with minimal changes in substrate affinity. Transformation of the model *V. vinifera* cv. Microvine 04C023V0006^47^ with the Muscat *VviDXS* N284 allele resulted in increase of monoterpene production in berries despite lower expression of the Muscat *VviDXS* N284 allele^46,48^. These findings suggesting that greater monoterpene production is mostly due to the higher catalytic efficiency of the N284 VviDXS variant.

Comparison of *VviDXS* haplotypes between Muscat and neutral varieties showed a reduced sequence diversity within the haplotypes carrying the N284 mutation, implying that the Muscat mutation arose once and has spread through selective pressure and breeding practices^43^. This is in agreement with studies showing that the Muscats are a distinct group descended from two progenitors: Muscat blanc and Muscat of Alexandria, itself the result of a cross between Muscat blanc and *V. vinifera* cv. Axina de tres bias^49–52^. Emanuelli et al. suggest a history of selection for Muscat flavor followed by crosses between Muscat and neutral cultivars to develop cultivars with other desired phenotypic characters^43^. The large number of direct descendants of Muscat blanc and Muscat of Alexandria supports this; the two cultivars are the direct parents of 58 and 255 cultivars, respectively, according to the *Vitis* International Variety Catalog (VIVC, www.vivc.de).

A number of cultivars unrelated to Muscats have been characterized for their Muscat flavor, e.g., Malvasias^49^, suggesting that other mutations within the MEP pathway may confer increased monoterpene biosynthesis. In addition to the major QTL containing *VviDXS*, numerous QTLs with smaller effects have been identified in marker-trait analyses of different mapping populations^40–42,53^. They may co-locate with downstream genes involved in regulation of monoterpene synthesis and metabolism.

## Monoterpene synthesis and modifications

With 69 putative genes identified, the terpene synthase (*TPS*) gene family is greatly expanded in *Vitis vinifera* and represents all of the angiosperm terpene synthase subfamilies except for *TPS*-*f*^14^. Seventeen *VviTPS* belonging to the *TPS-b* and *TPS-g* subfamilies have been functionally characterized as monoterpene synthases. The majority are multi-product enzymes^14^. In this section, we describe the genes functionally characterized as involved in the synthesis, i.e., VviTPS, and/or modification of the major monoterpenes geraniol, α-terpineol, and linalool.

**Fig. 1:**
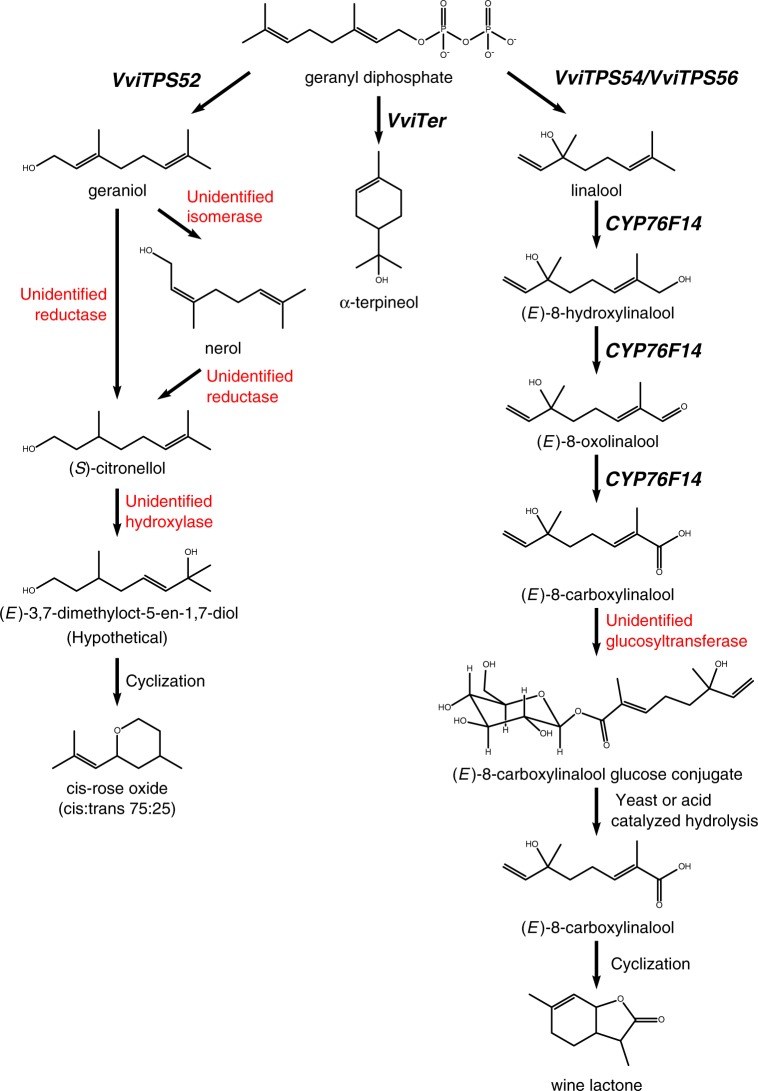
Proposed biosynthetic pathways of a selection of monoterpenes and monoterpene-derived metabolites in grapevine. Gene names are included where functionally characterized. (Adapted from refs. ^55,59,69^)

In aromatic cultivars, geraniol concentrations peak early in berry development followed by a decline until véraison, at which point the concentration increases dramatically^54^. *VviTPS52*, a geraniol synthase characterized by Martin et al.^14^, was shown to correlate with geraniol accumulation in ripening *V. vinifera* cv. Aleatico berries and to a smaller extent in Muscat blanc, indicating its importance in the biosynthesis of geraniol in ripe fruit^54^. Experiments with deuterium-labeled geraniol in *V. vinifera* cv. Scheurebe berries revealed that geraniol can be converted to nerol by an unknown isomerase. In addition, geraniol can be enzymatically reduced to (*S*)-citronellol by an unidentified reductase (Fig. 1). (S)-citronellol then undergoes hydroxylation and cyclization to form *cis* and *trans-*rose oxide^55^. *Cis*-rose oxide predominates in Gewürztraminer and Scheurebe wines and is a key component of the varietal character of Gewürztraminer^56–58^.

Although no enzymes responsible for the formation of rose oxides from (*S*)-citronellol have been identified, the process may occur in a similar manner as in rose geranium (*Pelargonium*) and roses (*Rosa damascena*)^55^. Wüst et al. proposed a mechanism of diol formation via a cytochrome oxidase with low specificity followed by acid-catalyzed cyclization of two of the four resulting diols as shown in Fig. 1^59,60^. Enantiomeric ratios of *cis-*rose oxides in grape, rose geranium, and rose all indicate the action of as yet unidentified enzymes. It is interesting to note that during fermentation, yeast can also produce both (−) and (+) enantiomers of *cis*-rose oxide from citronellol in sufficient quantities to impact wine aroma^61^. Therefore, the final impact of *cis*-rose oxide in wine depends not only on the amount synthesized by the grape but also on the level of citronellol present at harvest. The conversion rate of geraniol to citronellol increases in late berry development, indicating that the final stages of ripening are important in developing the characteristic aroma of Gewürztraminer and Scheurebe^55^.

As one of the more abundant monoterpenes in grape, α-terpineol is an important component of the character of aromatic varieties^23^. In addition to contributions from the rearrangement of other monoterpenes, α-terpineol is synthesized in the grape berry: a *TPS-b* subfamily α-terpineol synthase (*VviTer*) was one of the first monoterpene synthases identified and functionally characterized in grape^12^. A candidate gene association study of 61 grape cultivars found two SNPs in coding regions of the *VviTer* gene correlated with higher α-terpineol concentrations. These mutations result in non-synonymous amino acid substitutions (I38S and T519I); however, additional studies are needed to establish the sensory impact of these point mutations^62^.

Linalool is the predominant monoterpene in Muscats and plays a major role in their varietal aroma^58^. Despite having similar gain-of-function mutations in the active site of *VviDXS*, Muscat berries have a higher linalool content compared with Gewürztraminer^58^. While linalool is present in the berries of Gewürztraminer during the early developmental stages, concentrations decline rapidly as berries mature. In contrast, linalool concentrations in Riesling and Muscat blanc decrease initially followed by a very large increase after véraison^54,63^. QTLs associated with linalool content have been identified in the same region of LG 10 between SSR markers VrZAG 64 and VVIH01^42,53^. Duchêne et al. proposed a two-step model for Muscat aroma: one mutation in *VviDSX* conferring higher geranyl diphosphate (GPP) biosynthesis and another favorable allele near VrZAG64 shifting the monoterpene profile towards linalool^41^. No genes have been shown to co-locate with the QTL, but a number of possible candidates are located on chromosome 10.

One of the linalool synthases annotated by Martin et al.^14^ falls on chromosome 10, but most were located on unmapped scaffolds. The VCost.v3 annotation of the 12x.v2 genome assembly places many of the previously characterized linalool synthases on chromosome 10^64^. A (3S)-linalool synthase located on the chromosome, *VviTPS56*, has been shown to convert GPP to (3S)-linalool as a major product^14,63^. However, gene expression of *VviTPS56* in aromatic cultivars does not correlate with linalool accumulation during berry ripening^54,63^. On the other hand, *VviTPS54*, another linalool synthase, is expressed much more highly during the later stages of ripening (76–116 days after flowering) in Muscat blanc and Aleatico^54^. *VviTPS54* is still unmapped but has also been shown to produce (3S)-linalool from GPP in vitro and may be responsible for linalool synthesis in ripening berries^14,54^. Additional linalool synthases identified and characterized by Martin et al.^14^ as linalool/(E)-nerolidol/(E,E)-geranyl linalool synthases (*VviTPS57*, *58*, *61*, and *63*) have been proposed to be involved solely in nerolidol synthesis as they lack the plastid transit signal peptide characteristic of other monoterpene synthases^64^. Curiously, despite the abundance of annotated linalool synthases on chromosome 10, a genome-wide association study of 96 accessions composed of *V. vinifera*, *V. amurensis*, and *V. labrusca* cultivars and hybrids found QTLs associated with linalool content only on linkage groups 1, 5, 7, and 19^65^.

The oxidation products of linalool comprise a large proportion of the monoterpenoid diversity in wines^22^. Linalool is subject to a number of enzymatic reactions during fruit ripening that can generate monoterpenoids of higher oxidation states such as (*E*)-8-hydroxylinalool^66^. (*E*)-8-hydroxylinalool can then be further oxidized to (*E*)-8-carboxylinalool (Fig. 1). Under wine aging conditions (*E*)-8-carboxylinalool can undergo a slow acid-catalyzed cyclisation resulting in the production and accumulation of wine lactone, a bicyclic monoterpene lactone, over time^67^. With its extremely low odor threshold (10 ng/L), wine lactone is an important contributor to the aroma of white wines, particularly those made from Gewurztraminer, despite its low abundance (100 μg/L)^57^. Wine lactone is also found in some red wines^68^. Ilc et al. identified CYP76F14, a CYP76 enzyme highly expressed in ripe berries, that is capable of oxidizing linalool to (*E*)-8-carboxylinalool when expressed in *Saccharomyces cerevisiae* and *Nicotiana benthamiana*. While two other CYP76s expressed in ripening berries can also metabolize linalool, only CYP76F14 catalyzes the entire oxidative cascade from linalool to (*E*)-8-carboxylinalool^69^.

Marker-trait analysis of a Riesling x Gewurztraminer population showed that (*E*)-8-carboxylinalool concentration is associated with three major QTLs: one containing *VviDXS*, a QTL colocalizing with several terpene synthases that have since been assigned to chromosome 10, and finally one on chromosome 2 containing *CYP76F14*. These data provide support for the importance of CYP76F14 in (*E*)-8-carboxylinalool synthesis^69^. Ilc et al. also found no difference in the product profiles of CYP76F14 cloned from Muscat Ottonel and the non-aromatic *V. vinifera* cv. PN40024^69^. This, along with the slow conversion rate of (*E*)-8-carboxylinalool to wine lactone suggests that increased monoterpene production due to a *VviDXS* mutation is a prerequisite for wine lactone concentrations exceeding the sensory threshold. Presumably, some amount of linalool synthesis is required as well, although it is interesting to note that wine lactone occurs at suprathreshold levels in red wine cultivars that accumulate only modest amounts of linalool^68^. The (*E*)-8-carboxylinalool precursor has been detected in berries only as a glucose conjugate but no grape UDP glucosyltransferases have been identified that catalyze production of the glucoside^69,70^.

Glycosides represent the majority of the monoterpene pool in grapes. They can undergo hydrolysis during winemaking and aging and contribute to the aroma over time^11,27^. In grape, glycosides of aroma compounds occur as monosaccharides bound to a β-d-glucose moiety (glucosides) or as disaccharides consisting of glucose and rhamnose, apiose, or arabinose^27^. In *V. vinifera*, anthocyanins occur only as monosaccharides, suggesting that the glycosylation of aroma compounds in that species occurs through a different pathway^11^. Glycosyltransferases (GTs) are ubiquitous enzymes that play important roles in many metabolic processes. Although a large number of GTs have been identified in plants, only a small fraction has been functionally characterized. Grape GTs were among the first monoterpenol-specific GTs to be characterized^71^.

Three GTs, *VviGT7*, *VviGT14*, and *VviGT15* have been functionally characterized in *V. vinifera* and are expressed in conjunction with the accumulation of monoterpene glycosides in ripening berries^58,72,73^. All three enzymes share geraniol, nerol, and citronellol as major substrates and exhibit activity towards other aroma compounds known to occur as glycosides in berries such as lipoxygenase and phenylpropanoid pathway products. In addition, *VviGT14* is able to glucosylate linalool^72,73^. Li et al. found *VviGT14* to be particularly highly expressed in Muscat blanc, a cultivar with high linalool concentrations^58^. However, Bönisch et al. found that *VviGT15* was more highly expressed in Muscat blanc instead^72^. Expression of the three *GT*s has been shown to vary significantly between cultivars along with concentrations of free and bound monoterpenes, perhaps contributing to the differences in aromatic profile between cultivars, vintages, and locations^58^. Four alleles of *VviGT7*, two functional and two inactive, are present in Muscat blanc^73^. In contrast, *VviGT14* exists as a single functional allele in Muscat blanc and Gewürztraminer^58^. While these GTs are capable of glucosylating a wide variety of substrates, over 200 aroma compound glycosides have been isolated from grape and much remains to be learned about the genes involved in their formation^58,71–73^.

## Sesquiterpenes

Compared with monoterpenes, sesquiterpenes in grapes and wine have received less attention due to their lower volatility and higher detection thresholds^11,74^. In their review on the extraction and identification of grape-derived sesquiterpenes, Petronilho et al. recorded a total of 91 sesquiterpenes isolated from must, wines, distillates, and other grape products. 57 of these were identified from berries. Grape sesquiterpenes comprise predominately of hydrocarbons with some ketones, oxides, and alcohols^75^. Like monoterpenes, sesquiterpenes are synthesized from DMAPP and IPP, which in turn result from two parallel pathways, the cytosolic MVA and the plastidial MEP pathways. Although monoterpenes are largely synthesized from the MEP pathway, and sesquiterpene biosynthesis is thought to occur via the cytosolic MVA pathway, there is evidence of movement of intermediates from the plastid to the cytosol in a number of plants^25,28,76^.

The immediate precursor molecule for sesquiterpenes is farnesyl diphosphate, which is synthesized from one DMAPP molecule and two IPPs. In grape, both MVA-derived and MEP-derived DMAPP and IPP are incorporated into sesquiterpenes^76^. In Microvines transformed with the Muscat *VviDXS* N284 allele, sesquiterpene biosynthesis is increased in flowers compared with the wild type, albeit to a smaller extent than monoterpenes^48^. Since *VviDXS* is part of the MEP pathway and not the MVA pathway, this supports the possibility of some level of exchange between the two pathways. As of yet no genes encoding transporters involved in exchanging terpenoid intermediates have been identified.

Thirty putative sesquiterpene synthases have been identified from the PN40024 genome sequence^14^. The *VviTPS-a* subfamily represents a highly expanded group of predominantly sesquiterpene synthases on chromosomes 18 and 19. Thirteen members of the subfamily have been functionally characterized by Martin et al., and the majority of these synthesize multiple products from the FPP substrate^14^. However, the characterized sesquiterpene synthases have not been shown to produce the entire range of sesquiterpenes found in grape berries^25^. Biosynthesis of sesquiterpenes in berries appears to be restricted to the skin, which is consistent with their absence in the mesocarp and high concentration in the epicuticular wax of the exocarp^76^. Sesquiterpenes are also produced by grape anthers during bloom as pollinator attractants or herbivore deterrents. Martin et al. functionally characterized a (+)-valencene synthase (*VviVal*) expressed in grapevine flowers that produces (+)-valencene and (–)-7-epi-α-selinene as major products. Like many other TPS, *VviVal* expression decreases after bloom but increases again during late berry development in Gewürztraminer berries^12^. However, a survey of the sesquiterpene profiles of several varieties did not detect either product of *VviVal* in Gewürztraminer berries^77^.

The most prominent sesquiterpene identified from wine so far is rotundone. An important component of the aroma of *Piper nigrum*, rotundone is extraordinarily potent with detection thresholds of 16 ng/L in red wine and 8 ng/L in water^78^. Although notable as an impact compound in *V. vinifera* cv. Syrah wines where it can reach concentrations of 220 ng/L, rotundone has also been detected in wines made from a number of other varieties. Some other varieties contain relatively high levels of rotundone as well, such as *V. vinifera* cv. Durif, cv. Graciano, cv. Vespolina, and cv. Grüner Veltliner^74,79,80^. Rotundone accumulation begins at véraison and continues through the ripening period^80^.

**Fig. 2:**
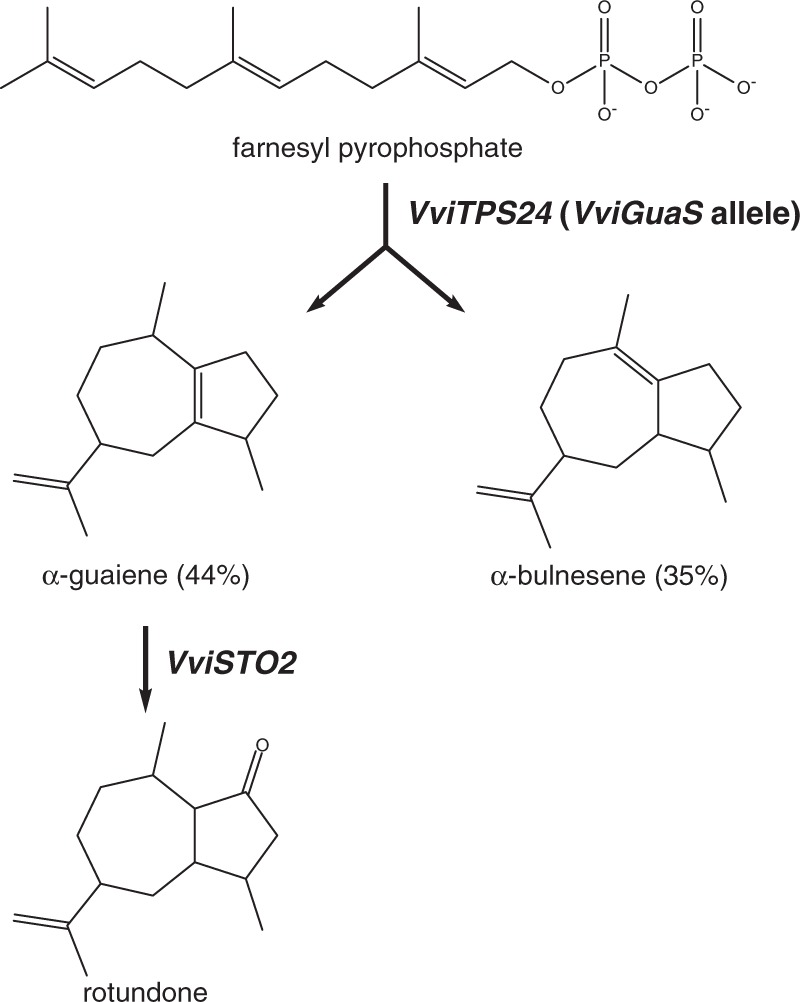
Formation of α-guaiene from farnesyl diphosphate (FPP) by the VviGuaS variant of VviTPS24 and formation of rotundone through the enzymatic oxidation of α-guaiene by VviSTO2, a member of the CYP71BE family of cytochrome p450 enzymes. (Adapted from refs. ^81,84^)

The precursor of rotundone is α-guaiene, a 5,7 bicyclic sesquiterpene found in high concentrations in Syrah berry skins, as well as in other plants that produce rotundone (Fig. 2)^81,82^. Concentrations of α-guaiene peak at 12 weeks post-flowering, while rotundone levels reach their maximum 14 weeks after anthesis^81^. Of the 13 sesquiterpene synthases functionally characterized by Martin et al., just one, *VviTPS24*, encodes an enzyme (VviPNSeInt) that produces α-guaiene and only as a minor product (3.5% of the total)^14^. *VviTPS24* is significantly upregulated during véraison in Syrah berries^83^. Drew et al. transformed *N. benthamiana* with *VviTPS24* cDNA isolated from Syrah berries and were able to detect α-guaiene and δ-guaiene as major products (44% and 35%, respectively)^84^. Due to the divergent range of products, the enzyme encoded by the Syrah cDNA was named VviGuaS. The transcript sequence of the VviGuaS variant of *VviTPS24* is 99.5% identical to that of the previously characterized VviPNSeInt variant. The amino acid sequences of the two gene products differ at six positions, two of which, corresponding to T414S and V530M substitutions in VviPNSeInt, are located in the active site.

Site-directed mutagenesis of VviGuaS cDNA altered the product profile towards that reported for VviPNSeInt, with the VviGuaS-T414S/V530M double mutant having the most similar profile to VviPNSeInt and very little α-guaiene production^84^. The VviGuaS variant of *VviTPS24* is absent from the PN40024 genome; however, the *V. vinifera* cv. Pinot Noir genome contains ambiguous base calls at four of the six positions that are polymorphic between the two variants of *VviTPS24*. This suggests that Pinot Noir is heterozygous for both VviGuaS and VviPNSeInt and that the VviGuaS allele was lost during breeding for homozygosity to produce PN40024^17,84^.

The existence of an allele with a major effect on the volatile profile of a cultivar and its absence in the reference genome illustrates a limitation of the PN40024 genome particularly as it derives from a genotype for which there is limited phenotypic data^18^. Recently, Smit et al. conducted a functional characterization of *VviTPS-a* genes expressed in the inflorescences of several *V. vinifera* cultivars and found cultivar-specific structural variations with major impacts on gene function and floral sesquiterpene profile^85^. It is likely that structural variations are also important contributors to differences in berry and wine aroma profiles. More research is needed to confirm if Pinot Noir is indeed heterozygous for the *VviGuaS* allele and to determine if Syrah is homozygous for the same allele. Heterozygosity of the *VviTPS24* gene in Pinot Noir, a variety with low levels of rotundone, implies the existence of other factors impacting the conversion of α-guaiene to rotundone.

Huang et al. demonstrated that α-guaiene undergoes aerial oxidation to rotundone^82^. However, *VviSTO2*, a cytochrome p450 enzyme located on chromosome 19 and belonging to the CYP71BE family, has also been shown to catalyze a one-step oxidation of α-guaiene to rotundone. *VviSTO2* is more highly expressed in berry skins than in the pulp and at much greater levels in Syrah than in *V. vinifera* cv. Merlot, a cultivar not known for accumulating rotundone. Expression is also concurrent with the accumulation of rotundone and peaks at 14 weeks after flowering. In vitro assays with VviSTO2 protein exhibited enzyme activity towards α-guaiene and (+)-valencene only. Since neither (+)-valencene nor its oxidation product β-nootkatol have yet been isolated from ripe Syrah berries and no other sesquiterpenes were utilized as substrates, it is likely that the primary role of *VviSTO2* is in rotundone production^81^. Low *VviSTO2* expression may account for the lack of rotundone in varieties with the VviGuaS variant of *VviTPS24*.

## Norisoprenoids

Norisoprenoids are a diverse group of widespread compounds derived from the oxidative breakdown of carotenoids^28,86^. They are important components of the aroma of many plants and plant products including roses, tomato (*Solanum lycopersicum*), tea (*Camellia sinensis*), saffron (*Crocus sativus*), watermelon (*Citrullus lanatus*), and osmanthus (*Osmanthus fragrans*)^87–89^. Although C_13_ norisoprenoids are most abundant in plants, compounds with 9 to 11 carbons occur as well^89^. A large number of norisoprenoids have been identified in grape berries and wine; however, only a few have been established to have sensory significance. As with monoterpenes, the majority are found in the berry as non-volatile glycosides^19,21,90^. Multiple precursors, each with various glycoside conjugates, are possible for each norisoprenoid^91^.

The most important norisoprenoids for wine aroma are β-ionone, β-damascenone, vitispirane, actinidol, 4-(2,3,6-trimethylphenyl)buta-1,3-diene (TPB), 1,1,6-trimethyl-1,2-dihydronaphthalene (TDN), and 2,2,6-trimethylcyclohexanone (TCH)^19^. In wine, TCH has been isolated only from Port and is the only C_9_ with significant sensory importance identified so far^92^. Since little is known about its biosynthesis, it will not be discussed further. β-ionone, a compound with a “woody” or “violet” aroma is widespread in plants and occurs around or above its detection threshold of 90 ng/L in the wines of many grape cultivars^93,94^. β-damascenone is ubiquitous in wines and many fruit products, and, despite only rarely exceeding detection thresholds, may have important indirect effects on wine aroma^95,96^. β-damascenone is also found in cultivars of *V. rotundifolia* and is particularly abundant (5 μg/kg) in *V. labrusca* cv. Concord^97^. TDN is characteristic of Riesling wines, while TPB was identified more recently. High levels of both TDN and TPB can contribute negative effects to wine aroma. The sensory contributions of vitispirane and actinidol are less clear^19^.

**Fig. 3:**
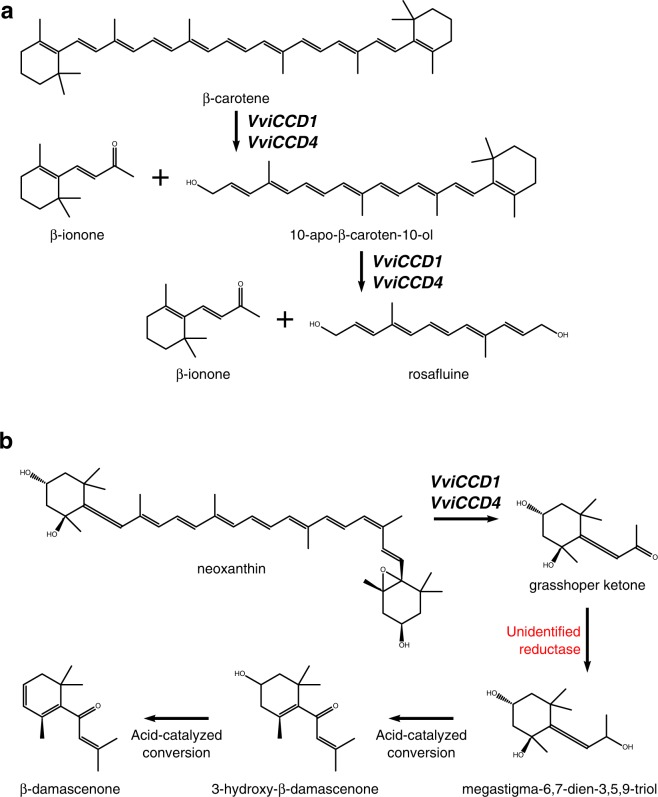
Formation of β-ionone and β-damascenone in grape. Cleavage of (**a**) β-carotene and (**b**) neoxanthin by 9,10,(9’,10’)-carotenoid cleavage dioxygenases (CCDs) and proposed subsequent formation of β-damascenone from the neoxanthin cleavage product grasshopper ketone. (Adapted from ref. ^19^)

Norisoprenoid formation from carotenoids can be non-enzymatic or catalyzed by a number of oxidative enzymes^86^. However, the abundance of C_13_ norisoprenoids in grapes, as well as the preservation of carotenoid asymmetric centers in norisoprenoid products suggests an enzymatic pathway^21,98^. The general mechanism of formation begins with cleavage of the carotenoid by a dioxygenase followed by enzymatic transformation of cleavage products to non-volatile precursors and ultimately conversion of the precursors to volatile norisoprenoids through acid catalyzed reactions (Fig. 3). Only β-ionone does not require further modification to be volatile^19^. Norisoprenoids and their precursors are subject to a large number of possible reactions that adds enormous complexity to the study of their biosynthesis^21^. The carotenoid precursors of norisoprenoids are extremely important pigments with essential roles in many plant functions such as photosynthesis, flower and fruit color, and phytohormone biosynthesis^28^. While carotenoids play a major role in the skin color of many ripe fruits, the carotenoid profile in grape berries is more similar to that of leaves than other fruit^99^. During berry ripening, there is a simultaneous increase in norisoprenoid content and decrease in carotenoid content^98^.

Like monoterpenes, carotenoids are synthesized in the plastids via the MEP pathway^21^ and share DXS as a rate-limiting enzyme in their biosynthesis. Nonetheless, there have been limited studies comparing the carotenoid or norisoprenoid levels between grape cultivars with N284 and K284 *VviDXS*^48,100^. Although, overexpression of K284 *VviDXS*, as well as transformation with the Muscat N284 *VviDXS*, causes upregulation of some genes involved in carotenoid production during anthesis and véraison, the effect on carotenoid content is unclear. Conversely, the overexpression of *E. coli DXS* in tomato results in greater accumulation of carotenoids and chlorophylls^101^.

The initial cleavage of carotenoid molecules to yield C_13_ norisoprenoid skeletons is carried out by 9,10,(9’,10’)-carotenoid cleavage dioxygenases (CCDs, Fig. 3). Although, nine *CCD* members have been identified in *Arabidopsis*, only CCD1 and CCD4 have been shown to cleave carotenoids at the 9,10 and 9’,10’ double bonds to yield two C_13_ ketone end groups and one C_14_ aldehyde. *CCD4*s have been functionally characterized in *Crocus sativus*, *Malus x domestica*, and *Chrysanthemum x morifolium* and cleave β-carotene to give β-ionone^88,102,103^. Mathieu et al. identified *VviCCD1* from a Syrah EST database. When expressed in *E. coli*, the recombinant protein catalyzed the cleavage of zeaxanthin and lutein to form 3-hydroxy-β-ionone but did not generate any cleavage products with β-carotene^100,104^. In addition, unlike plastid-localized CCD4s, CCD1s are found in the cytosol where their carotenoid substrates are much less abundant^105^. Nevertheless, the strong correlation between *CCD1* expression and volatile C_13_ norisoprenoid production in rose and petunia (*Petunia* x *atkinsiana*) suggests that the gene plays a role in the production of norisoprenoids in those flowers^106,107^.

Overall, 72 non-redundant genes putatively involved in carotenoid metabolism in *V. vinifera* were identified by Young et al. and Leng et al.^15,108^. Of these, seven were annotated as CCDs: *VviCCD1.1, VviCCD1.2, VviCCD4a, VviCCD4b, VviCCD4c, VviCCD7*, and *VviCCD8*. In Pinot noir, *VviCCD1* exists as a tandem duplication on chromosome 13, while there are three paralogues of *VviCCD4* located on chromosomes 2 and 16 with highly conserved amino acid sequences^15,16^. Functional characterization of *VviCCD1*, *VviCCD4a*, and *VviCCD4b* isolated from *V. vinifera* cv. Pinotage determined that they were all able to catalyze 9,10(9’,10’) cleavage from a variety of carotenoid substrates, with VviCCD1 being the sole CCD to produce β-ionone from β-carotene^16^. The expression patterns of *VviCCD1*, *VviCCD4a*, and *VviCCD4b* supports the possibility of their role in norisoprenoid biosynthesis.

*VviCCD1* expression has been found to increase several fold at véraison in a number of grape cultivars, while *VviCCD4a* and *VviCCD4b* expression peaks during ripening^15,16,104,108^. *VviCCD1* and *VviCCD4* transcript levels are negatively correlated with carotenoid content in Fujiminori, a tetraploid table grape with *V. vinifera* and *V. labrusca* parentage, and Sauvignon blanc^15,108^. Similarly, norisoprenoid content peaked 1 week after induction of *VviCCD1* expression in Muscat of Alexandria and Syrah^100^. Norisoprenoid profiles of the two cultivars were dominated by modified (via an oxidase or reductase) products of carotenoid cleavage. A grape cell suspension culture was also shown to metabolize β-ionone into oxygenated, reduced, and glycosylated derivatives, but the enzymes responsible have not yet been identified^109^. On the other hand, the direct cleavage product of zeaxanthin and lutein by VviCCD1, 3-hydroxy-β-ionone, is a major component of the free norisoprenoids in Muscat of Alexandria^100^.

Although the preceding in vitro experiments and expression patterns suggest a role for VviCCD1 in the cleavage of carotenoids, transformation experiments give a more complicated picture. Overexpression of *VviCCD1* in *V. vinifera* cv. Sultana did not affect leaf carotenoid nor norisoprenoid concentrations^16^, while in tomato (*Solanum lycopersicum*), silencing of *LeCCD1* resulted in a 50% decrease fruit β-ionone concentrations without reducing carotenoid levels^110^. When incubated in vitro with various carotenoids and apocarotenoids, *OsCCD1*-overexpressing rice (*Oryza sativa*) endosperms metabolized apocarotenoids more effectively than carotenoids^111^. Ilg et al. proposed that the apocarotenoid cleavage activity observed with OsCCD1 is due to a function of CCD1s as scavengers of non-enzymatic cleavage products of carotenoids resulting from high light stress^111^. Similarly, the role of VviCCD1 may be to catalyze the cleavage of apocarotenoids that have been exported into the cytoplasm after partial metabolism by other CCDs such as CCD4s, whose expression is upregulated during berry development^16^.

Unlike β-ionone, β-damascenone is not the direct product of dioxygenase cleavage of a carotenoid. Concentrations in berries are generally much lower than in wine, indicating that further chemical transformations are necessary to generate free β-damascenone^96^. The formation of β-damascenone is complex and has been reviewed in detail by Sefton et al.^96^. A major proposed precursor of β-damascenone is grasshopper ketone, a ketodiol resulting from dioxygenase cleavage of neoxanthin^19,112^. The ketodiol is then enzymatically reduced to megastigma-6,7-dien-3,5,9-triol which may then undergo acid-catalyzed conversion to β-damascenone (Fig. 3b)^89,96,113^. The two compounds have been isolated from grape juice as glycoside conjugates, supporting their role as the precursors for β-damascenone formation^114,115^. While no candidate enzymes have been proposed for the reduction of grasshopper ketone to form the triol, a rose CCD1 (RdCCD1) has been shown to produce grasshopper ketone from neoxanthin^107^. It remains to be determined if any VviCCDs have the same activity.

In contrast to the fairly ubiquitous β-ionone and β-damascenone, only a few genotypes produce wines with aromatically significant quantities of TDN. TDN is a characteristic component of Riesling wines and reaches concentrations as high as 200 μg/L^116,117^. Very little research on TDN content in cultivars besides Riesling has been carried out, but small amounts of TDN have been identified from juices and wines of other cultivars^117,118^. As TDN is produced from the slow hydrolysis of glycosylated precursors during wine fermentation and aging^116,119^, this suggests that Riesling has higher concentrations of TDN precursors than other cultivars; however, the precursors are not completely absent in other grapes. Although a number of possible precursors have been isolated, neither the enzymes involved, nor the starting carotenoids have been identified.

Compared with the other major norisoprenoids, the formation of vitispiranes, actinidols, TPB, and their precursors is still poorly understood. TDN concentrations have been found to correlate positively with vitispirane, although it is possible that TDN can be reduced to the latter compound by yeast during fermentation^120^. As with TDN, a number of norisoprenoid precursors are possible for TPB. Three, including the actinidols, occur as glycoconjugates and are hydrolyzed to form TPB during wine aging^121^. It is interesting that while Riesling, a cultivar with moderate levels of monoterpenes, has an abundance of TDN and vitispirane, Muscat cultivars rich in monoterpenes do not. Riesling is also not significantly higher in β-damascenone or β-ionone^96^. This suggests the existence of genetic mechanisms not found in other cultivars that increase the formation of TDN and vitispirane precursors.

## Methoxypyrazines

3-alkyl-2-methoxypyrazines are a widespread class of potent odorants found in many kingdoms^122–124^. They are often characterized by extremely low detection thresholds (2–16 ng/L in wine), as well as by aromas described as herbaceous, green, vegetal, and earthy^123,125^. Methoxypyrazines have been detected in a number of *Vitis* species such as *V. amurensis, V. cinerea, V. riparia, V. rupestris*, and *V. vinifera*^2,3^. Their high concentration in unripe fruit, toxic plants, and aposematic insects suggests a protective role for developing seeds^126^. These compounds constitute an integral element of the aromas of some French *V. vinifera* cultivars such as Cabernet Sauvignon and Carménère^11,123^. Smaller quantities of methoxypyrazines have also been found in other cultivars: Grenache, Pinot noir, Riesling, and Syrah^24,127^. Although they can be perceived negatively when present in excess in wine, methoxypyrazines are desirable in small quantities, particularly for white wines^11^. The most abundant methoxypyrazine in grapes and wine is 3-isobutyl-2-methoxypyrazine (IBMP), a compound also responsible for the characteristic aroma of green bell pepper. Unlike IBMP, the next two most abundant methoxypyrazines, 3-isopropyl-2-methoxypyrazine (IPMP) and 3-sec-butyl-2-methoxypyrazine (SBMP), rarely occur above detection threshold in grape berry^123^.

Methoxypyrazines reach their highest concentrations in the vegetative organs of the grapevine. In contrast to its low abundance in berries, IPMP reaches concentrations of 8000 ng/L in roots. On the other hand, IBMP is most abundant in mature basal leaves^123^. In berries, methoxypyrazines show a distinct pattern of accumulation in early development until the onset of véraison followed by rapid decrease until harvest regardless of final concentration^124,127,128^. For varieties with low methoxypyrazine levels in mature fruit, prevéraison concentrations are a fraction of those in high-methoxypyrazine varieties and decline to undetectable levels after véraison^127,129^. The mechanisms for this decline have not been resolved and may be due to a dilution effect from berry expansion or demethylation to a nonvolatile hydroxypyrazine^124,127^.

A number of biosynthetic pathways have been postulated for the production of methoxypyrazines. A mechanism for bacterial synthesis starting with amidation of a branched chain amino acid followed by condensation with glyoxal was first proposed in 1970. However, the reaction is chemically unfavorable and glyoxal has not been isolated in plants^130,131^. A second pathway involving the condensation of two amino acids to form the cyclic backbone has been proposed as well, but neither have been demonstrated in plants^123,132^. Only the final *O*-methylation of the nonvolatile 3-alkyl-2-hydroxypyrazine precursors has been elucidated in grapevine^123^. 3-isobutyl-2-hydroxypyrazine (IBHP) occurs in comparable concentrations in pre-véraison berries of low-methoxypyrazine and high-methoxypyrazine varieties, indicating that differences in accumulation are not due to a lack of precursor molecules^127,133^.

Methylation of the precursors is catalyzed by *O*-methyltransferases (OMTs), which use *S*-adenosyl-L-methionine as the methyl donor. Four *OMT*s have been identified in grapevine (*VviOMT1, VviOMT2, VviOMT3*, and *VviOMT4*)^129^. The first grape OMT with activity towards hydroxypyrazines was isolated in 2001 from Cabernet Sauvignon berries^134^. The gene encoding this OMT was subsequently identified by Dunlevy et al. as *VviOMT1* along with another *OMT* with a similar gene sequence (*VviOMT2*). Recombinant *VviOMT1* and *VviOMT2* products exhibited activity towards hydroxypyrazine substrates with VviOMT1 having greater catalytic efficiency. However, hydroxypyrazines may not be their main substrate, as both enzymes had 100-fold greater activity towards caffeic acid and quercetin^134,135^. To identify additional loci affecting methoxypyrazine accumulation, QTL analysis was performed on several mapping populations consisting of progeny between Cabernet Sauvignon, a high-IBMP cultivar, and a low-IBMP producing parent^129,133^.

Analyzing IBMP production in an F_1_ population derived from Cabernet Sauvignon and a dwarf vine derived from *V. vinifera* cv. Pinot Meunier showed that all progeny produced IBMP at varying concentrations. This lead Dunlevy et al. to hypothesize that production of IBMP is a dominant trait for which Cabernet Sauvignon is homozygous. A 3:1 segregation ratio of IBMP production in a derived F_2_ population confirmed their hypothesis. In addition, a methoxypyrazine-associated QTL was located on chromosome 3 in the F_2_ population. This locus contained 261 putative genes from which *VviOMT3* and *VviOMT4* were identified^133^. Guillaumie et al. conducted a separate marker-trait association on a population of Cabernet Sauvignon x *V. riparia* cv. Gloire de Montpellier. They identified five QTLs accounting for 41% of the variance in IBMP content, including the QTL encompassing *VviOMT3* and *VviOMT4* along with another QTL colocalizing with *VviOMT1* and *VviOMT2*^129^.

Functional characterization of *VviOMT3* and *VviOMT4* by Dunlevy et al. found that Cabernet Sauvignon VviOMT3 has a catalytic efficiency 150-fold to 7000-fold greater than VviOMT1, 2, and 4. Interestingly, VviOMT3 encoded by an allele from the low-methoxypyrazine cultivar Pinot noir was found to be only slightly less efficient than Cabernet Sauvignon, which is in contrast to Guillaumie et al.’s findings^129,133^. VviOMT3 is relatively specific for hydroxypyrazines, and substrate specificity between alleles from different cultivars is not significantly different^129,133^. Together with the similar catalytic efficiency of VviOMT3 alleles from low-methoxypyrazine varieties, this suggests that varietal differences in methoxypyrazine levels is not due to altered enzyme function but instead due to variations in gene expression.

Several studies have shown that the expression of *VviOMTs* peaks early in berry development^124,129,133,136^. A lack of differences in *VviOMT1* and *VviOMT2* transcript levels between high-methoxypyrazine and low-methoxypyrazine cultivars suggests that these enzymes are not responsible for differences in IBMP concentration, but may produce the small amount measured in low-methoxypyrazine cultivars. In contrast, *VviOMT3* is many orders of magnitude more highly expressed in Carménère and Cabernet Sauvignon than in Pinot noir or Petit Verdot^129,133^. The upregulation of *VviOMT3* in high-methoxypyrazine genotypes and its lack of expression in other genotypes suggests that differences in transcription are responsible for variations in methoxypyrazine content.

To determine the mechanisms associated with *VviOMT3* expression variation, Dunlevy et al. examined the non-coding regions of the gene in Cabernet Sauvignon and Pinot noir^133^. Three alleles were identified: one for which Cabernet Sauvignon is homozygous, and two in Pinot noir. Although the exact mechanism suppressing *VviOMT3* expression in Pinot noir is not yet known, the authors identified polymorphisms among the three alleles including a transposon in one Pinot noir allele. All Cabernet Sauvignon × Pinot Meunier F_2_ progeny that contain at least one Cabernet Sauvignon *VviOMT3* allele accumulate methoxypyrazines. This indicates that a single Pinot allele is insufficient for repressing the Cabernet Sauvignon allele. Association mapping of 91 *V. vinifera* cultivars found that cultivars with the highest methoxypyrazine concentrations possess at least one copy of the Cabernet Sauvignon *VviOMT3* allele^133^. Interestingly, Cabernet Sauvignon, Carménère, and Merlot are all high-methoxypyrazine cultivars, suggesting a positive selection for this distinctive aroma trait likely inherited from their common parent Cabernet Franc^137^.

## Furan derivatives

Furanones are pentose-derived and hexose-derived odorants, often with extremely low odor detection thresholds, that constitute important components of the aromas of some fruits. The most prominent of these is 4-hydroxy-2,5-dimethyl-3(2H)-furanone or furaneol, a compound described as having a strawberry or caramel aroma at low and high concentrations, respectively^28^. Furaneol is the most abundant compound in the free and bound volatile profiles of *Muscadinia rotundifolia* and a major component of the characteristic aroma of *V. labrusca*. The majority of furaneol in *M. rotundifolia* berries exists as non-volatile furaneol glucopyroside^26^. Ninety five percent of the furaneol content and 72% of furaneol glucopyranoside in berries of the interspecific hybrid cultivar Muscat Bailey A are contained in the flesh^138^. The most common furan derivatives in *V. vinifera* wine, e.g., furfural and sotolon, are introduced during fermentation and aging^11^. However, furaneol has also been identified from the wines of a number of *V. vinifera* cultivars. Concentrations are much lower than in *M. rotundifolia* and *V. labrusca* hybrids, but can still significantly exceed the detection threshold of 5–37 μg/L^56,139,140^. Low concentrations of furaneol may contribute to the fruitiness of *V. vinifera* wines^140,141^.

The biosynthetic pathway for furaneol has not been elucidated in grape, and only a few key enzymes have been functionally characterized in strawberry (*Fragaria* x *ananassa*) and tomato^142^. In strawberry, D-fructose-1,6-diphosphate is first converted into 4-hydroxy-5-methyl-2-methylene-3(2H)-furanone (HMMF) by a yet unknown pathway^143^. HMMF is then converted into furaneol by an enone oxidoreductase (*FaEO*). An enone oxidoreductase bearing 71% amino acid identity with *FaEO* was identified in tomato and shown to have similar activity towards HMMF when expressed in *E. coli*. This, along with an enone oxidoreductase identified in furaneol-rich pineapple (*Ananas comosus*), suggests that the furaneol biosynthetic pathway may occur in *Vitis* as well^142,144^.

**Fig. 4:**
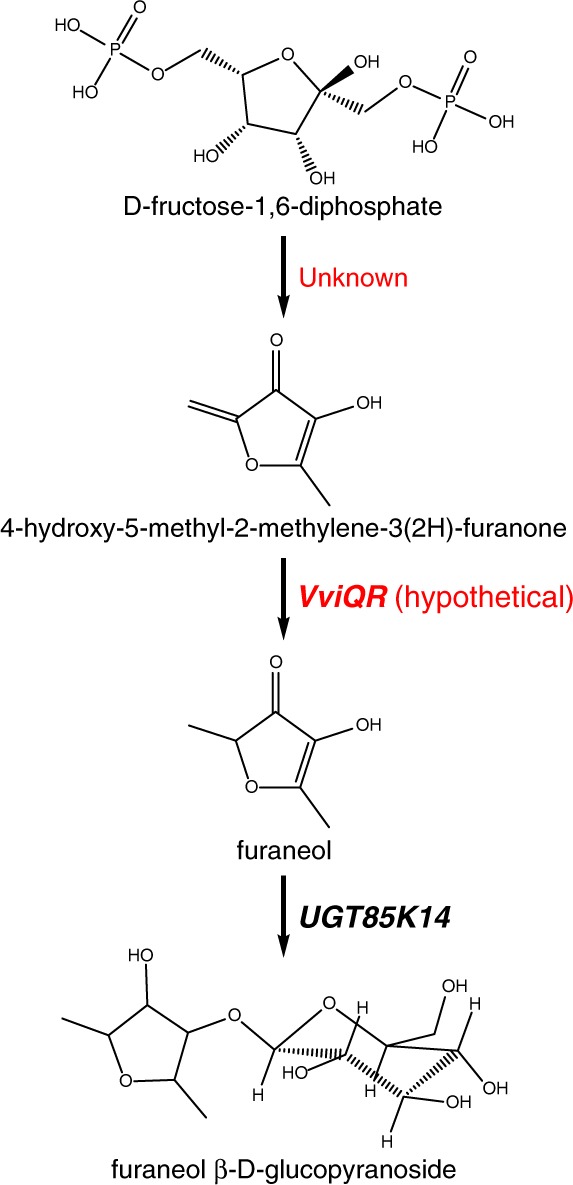
Putative biosynthetic pathway of 4-hydroxy-2,5-dimethyl-3(2H)-furanone (furaneol) from D-fructose-1,6-diphosphate and subsequent glucosylation by UDP-glucosyltransferase UGT85K14. (Adapted from refs. ^144,145^)

An enone reductase sharing 73% identity with FaEO was identified among the PN40024 predicted proteins but has not yet been shown to catalyze furaneol formation from HMMF^145^. Like strawberries, *V. labrusca* hybrids accumulate large amounts of furaneol glucoside. Small amounts of the glucoside are found in *V. vinifera* berries as well and can act as precursors for furaneol during wine fermentation and aging^138^. A UDP-glucose:furaneol glucosyltransferase (*UGT85K14*) with 98% sequence identity between *V. labrusca* and *V. vinifera* was identified from Muscat Bailey A by Sasaki et al.^145^. Heterologous expression of *UGT85K14* produced a protein that catalyzed the production of furaneol glucoside from furaneol and UDP-glucose (Fig. 4). Despite the major differences in furaneol and furaneol glucoside concentrations between *V. labrusca* and *V. vinifera* cultivars, *UGT85K14* is expressed in berries from both species and expression is not significantly different between *V. labrusca* cv. Concord and Cabernet Sauvignon^145^.

## Lipoxygenase pathway products

The products of the lipoxygenase pathway, primarily C_6_ and C_9_ aldehydes and alcohols, are involved in plant wound responses and defense strategies against pests and diseases^28^. They comprise a major percentage of total grape and wine volatiles, ranging from 40 to 97% in *V. vinifera* and *V. amurensis* cultivars and between 25 and 57% in *V. labrusca* cultivars and hybrids^2,146–148^. Lipoxygenase products are often described as having an herbaceous, leafy “green” aroma and can be a negative contributor to wine aroma^149,150^. However, they contribute a non-negligible supply of substrates necessary for the formation of desirable esters during fermentation^150^. C_6_ aldehydes may also be involved in the production of volatile thiols, as described in the following section. Like many volatile compounds found in grapes, a large proportion of C_6_ compounds are glycosylated. Functionally characterized monoterpenol glucosyltransferases (GTs) were shown to exhibit in vitro catalytic activity towards C_6_ alcohols, but it is unknown if they have this role in vivo^72^. In many plants, the lipoxygenase pathway is activated upon tissue damage or pathogen infection; however, the presence of C_6_ compounds in undamaged ripe berries suggests that the pathway is active to some extent^34^.

**Fig. 5:**
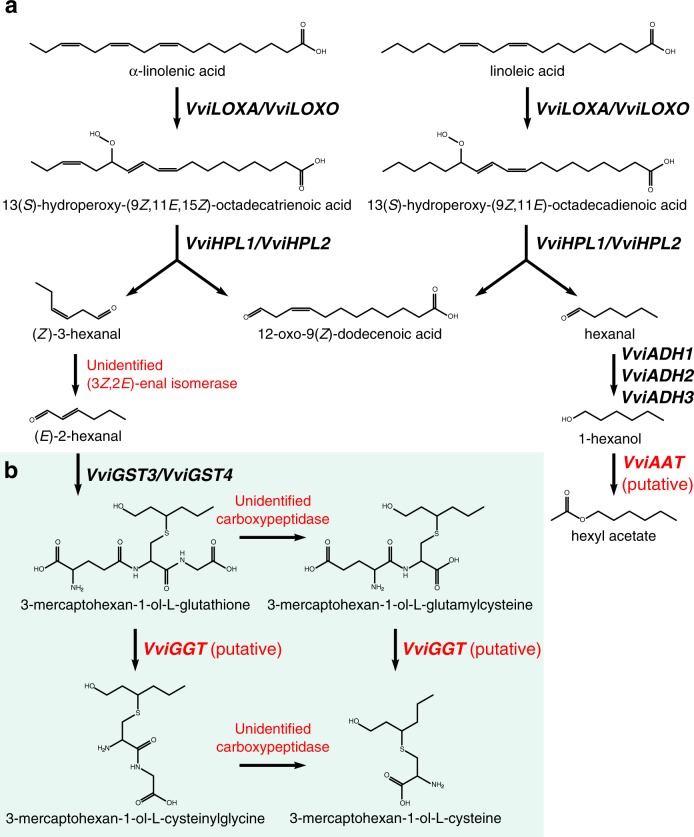
Proposed biosynthesis of volatile lipoxygenase pathway products and polyfunctional thiol precursors. **a** Schematic representation of lipoxygenase pathway metabolism of free fatty acids in grape berries. Not all products are shown. Conversion of 1-hexanol to hexyl acetate by VviAAT is putative and has not been shown experimentally. **b** Hypothetical 3-mercaptohexan-1-ol precursor formation from E-2-hexenal. Only VviGST3 and VviGST4 have been functionally characterized (Adapted from^21,152,155,180^)

The production of C_6_ alcohols and aldehydes from free fatty acids is initiated by lipoxygenases (LOXs, Fig. 5a). LOXs are a ubiquitous family of fatty acid dioxygenases that target polyunsaturated fatty acids such as linoleic and α-linolenic acids. Plant LOXs can be classified into two groups by their target oxygenation site at either the 9^th^ (9-LOXs) or 13th (13-LOXs) carbon of the fatty acid. 13-LOXs can be further classified by the presence (Type II) or absence (Type I) of a plastidic transit peptide^28,151^. Podolyan et al. identified 12 potential genes from the PN40024 reference genome based on sequence similarity with known plant LOXs^152^. Four of the identified LOXs, two Type II 13-LOXs (*VviLOXA* and *VviLOXO*) and two 9-LOXs (*VviLOXC* and *VviLOXD*), are expressed in Sauvignon blanc berries. Expression patterns of the four LOX genes vary across berry tissue type and development and are affected by wounding and botrytis infection^152^. Recombinant VviLOXA and VviLOXO proteins were shown to oxygenate unsaturated fatty acids in an in vitro assay, yielding two hydroperoxides: 13(*S*)-hydroperoxy-(9*Z*,11*E*,15*Z*)-octadecatrienoic acid (13(*S*)-HPOT) from α-linolenic acid and 13(*S*)-hydroperoxy-(9*Z*,11*E*)-octadecadienoic acid(13(*S*)-HPOD) from linoleic acid (Fig. 5a)^152^.

Hydroperoxides then undergo cleavage by hydroperoxide lyases (HPLs) to form C_6_ and C_9_ aldehydes and oxoacids^28^. HPLs are CYP74B/C cytochrome P450 enzymes that act on the hydroperoxy functional group and can be divided into three classes based on their substrate specificity. 9-HPLs and 13-HPLs are specific to 9(*S*)-hydroperoxides and 13(*S*)-hydroperoxides, respectively, while 9/13-HPLs are capable of catalyzing cleavage of both hydroperoxides^21,28^. *VviHPL1* and *VviHPL2*, located on chromosomes 12 and 3, respectively, catalyze the cleavage of (13(*S*)-HPOD) into hexanal and 13(*S*)-HPOT to form (*Z*)-3-hexenal and (*E*)-2-hexenal in *V. vinifera*. The latter product is likely the result of isomerization of (*Z*)-3-hexenal either via a 3*Z*,2*E*-enal isomerase or spontaneous rearrangement^28^. Although as of yet uncharacterized in grape, 3*Z*,2*E*-enal isomerases responsible for converting (*Z*)-3-hexenal to (*E*)-2-hexenal have recently been characterized in cucumber (*Cucumis sativus*) and pepper (*Capsicum annuum*)^153,154^.

While VviHPL1 is restricted to 13(*S*)-hydroperoxides, VviHPL2 is also capable of utilizing 9(*S*)-hydroperoxides as a substrate, producing C_9_ aldehydes. VviHPL1 has a much higher catalytic activity towards 13(*S*)-HPOT compared with VviHPL2^155^. In several cultivars, both genes are expressed at a low level until véraison when they increase rapidly in expression followed by a peak near maturity. Expression of both VviHPL genes correlates with the accumulation of hexanal and the two hexenals until the 10th week post-flowering, after which their concentrations decrease while HPL expression continues to increase^1,34,155,156^. The predominance of (*E*)-2-hexenal over hexanal immediately after véraison in Cabernet Sauvignon led Kalua and Boss to suggest that the α-linolenic acid to 13(*S*)-HPOT to (*E*)-2-hexenal pathway is favored^34^. Decreases in C_6_ aldehydes during fruit ripening may be due to conjugation with glutathione or reduction to alcohols by alcohol dehydrogenases (ADHs)^28,157^.

Like in many other angiosperms, grape ADHs are encoded by a small multi-gene family^158–160^. Six ADH isoforms, *VviADH1-6*, have been identified on chromosomes 4 and 18. Only *VviADH1-3* have so far been shown to be expressed in berries^161,162^. *VviADH1* and *VviADH3* increase in expression from fruit set until véraison upon which expression levels decline^163^. This is consistent with the accumulation of aliphatic alcohols in berries of the interspecific hybrid Kyoho^1^. In contrast, concentrations of hexanol in Cabernet Sauvignon berries increase dramatically at véraison and continue increasing through ripening^34^. This difference in alcohol accumulation may be due to their utilization as substrates for ester production in Kyoho.

Unlike *VviADH1* and *VviADH3*, *VviADH2* shows very low expression prior to véraison followed by a rapid increase in expression and enzyme activity. At its peak, *VviADH2* expression is considerably higher than the maxima of the other two ADH isoforms^1,156,163,164^. In a number of varieties, *VviADH2* is downregulated in late ripening; however, enzyme activity continues to increase, indicating that there is little turnover in the enzyme^163^. Although grapes are a non-climacteric fruit, the promoter of *VviADH2* contains ethylene response elements, and ADH activity is increased in *V. vinifera* cells grown in suspension in the presence of ethylene-generating compounds^161^. Despite significantly altered ADH activity, under-expression and overexpression of *VviADH2* in *V. vinifera* cv. Portan caused minimal changes to the concentrations of volatile compounds in mature berries^164^. This is in contrast to similar experiments in tomato where increased ADH activity resulted in higher concentrations of hexanol and (*Z*)-3-hexenol^165^.

As the most highly expressed *VviADH* in ripe berries, *VviADH2* may also be important for the production of wines through carbonic maceration^166^. ADH is necessary for the regeneration of NAD^+^ during alcoholic fermentation, and the upregulation of *ADH* is a common response to anaerobic conditions in plants^167,168^. Although research on gene expression during anaerobic metabolism of grapes is limited, anaerobic conditions were shown to induce the production of ADH protein in cultured cells but not whole berries^169^. Carbonic maceration has been shown to increase the concentration of higher alcohols in grape berries^170,171^. This may be due to the action of VviADH2 after the onset of anaerobic metabolism. More research is necessary to elucidate the role of VviADH2 in the development of volatile compounds associated with carbonic maceration.

Alcohols produced by ADH can contribute to grape and wine flavor on their own or as substrates for ester production by endogenous and yeast alcohol acyltransferases (AAT)^150^. Despite their predominance in wine, most wine esters are the product of yeast metabolism^172^. Endogenously produced esters are not an important component of ripe *V. vinifera* berries. In contrast, at 33% to 73% of total volatile compounds, esters constitute a major proportion of the volatile profile of *V. labrusca*, *V. riparia* and *M. rotundifolia* berries^26,148,173^. The predominant esters in *V. labrusca* and *M. rotundifolia* cultivars and hybrids are ethyl acetate, ethyl butanoate, ethyl 2-butenoate, ethyl hexanoate, and ethyl 2-hexenoate^1,26,148^.

Like many other aroma compounds, esters accumulate in berries beginning at véraison and rapidly increase in concentration until ripening. Ji et al. found that the rapid post-véraison increase in ester concentration coincided with dramatic upregulation of *VviAAT* expression in Kyoho^1^. Although ester concentrations in *V. vinifera* berries are typically very low, *VviAAT* expression and ester accumulation during berry development parallels that of Kyoho in some cultivars, albeit at lower amounts and with a predominance of C_6_-acetates^156^. Concentrations of (*Z*)-3-hexenyl acetate and (*Z*)-3-hexenyl butanoate are comparable with those of monoterpenes during the early development of Cabernet Sauvignon berries. However, ester concentrations decline to low levels after véraison. This suggests that VviAAT is present only during early development^34^.

VviAAT has not yet been experimentally shown to have enzymatic activity^8^. AATs belong to the BAHD superfamily of acyltransferases. They share the ability to act on multiple alcohols and acyl-coAs, producing a wide range of products^28,174^. AATs in kiwi (*Actinidia chinensis*) and apple can utilize a range of substrates deriving from the lipoxygenase pathway and amino acid metabolism with the products determined by enzyme specificity and relative substrate concentrations^175,176^. The only studied grape *AAT* encodes an anthraniloyl-coenzyme A:methanol acyltransferase (AMAT) and was isolated from *V. labrusca* cv. Concord. This *AMAT* bears 81% sequence similarity to *VviAAT*^8^. AMAT catalyzes the production of methyl anthranilate, a compound responsible for “labrusca character,” from anthraniloyl-coA and methanol. The enzyme has been shown to utilize multiple different alcohols as a substrate and may also play a role in the biosynthesis of butanoate and hexanoate esters in *V. labrusca* berries^8^. However, a study on the inheritance of “labrusca character” found no correlation between methyl anthranilate and total ester concentrations^177^. Methyl anthranilate is an important component of the varietal character of *V. labrusca* cultivars; however, it is derived from the phenylpropanoid pathway and will be further discussed in the corresponding section.

## Volatile sulfur compounds

Volatile sulfur compounds range from intensely malodorous to pleasantly fruity and are integral to the aromas of many plants such as passionfruit, black currant, garlic, and asparagus. They are also important volatile components of meat, mushrooms, and wine^11,21,178^. Until the discovery of thiols that contribute positive aromas to wines, sulfur compounds were generally associated with wine defects^178^. The most common volatile sulfur compounds in wine are thiols, hydrogen sulfide, dimethylmercaptans, and methylthioesters. Many of these are the products of microbial metabolism during fermentation; however, polyfunctional thiols and methylthioesters are crucial components of “varietal character” in some varieties and originate from precursors found in the berry. Sauvignon blanc is most well-known for aroma characters derived from thiols, but sulfur compounds are also important contributors to *V. vinifera* cultivars such as Muscat blanc, Gewürztraminer, Riesling, Chenin blanc and Semillon^11,179^. Polyfunctional thiols and their precursors have been previously reviewed in excellent detail^20,178,180^. Aside from a brief overview on the most important thiols and their precursors, this section will focus on their biosynthesis and what little is known about the genes involved.

4-mercapto-4-methylpentan-2-one (4MMP), a compound with the aroma of box tree (*Buxus*), was the first sulfur compound identified as an important contributor to the aroma of Sauvignon blanc^181^. Subsequently, Tominaga et al. identified 3-mercaptohexan-1-ol (3MH) and its acetate ester (3MHA), often described as “grapefruit” and “passionfruit”, respectively, from Sauvignon blanc wine^182,183^. Although generally present at lower concentrations, 3MH and 3MHA were found to also contribute to perceived fruitiness in wines made from Cabernet Sauvignon and Merlot^184,185^. 4-mercapto-4-methylpentan-2-ol (4MMPOH) contributes to the citrus elements of the aromas of Muscat blanc and Sauvignon blanc^179,183^. These four compounds represent the major contributors to Sauvignon blanc character and have perception thresholds of 0.8–60 ng/L^20^. Compounds such as 2-furanmethanethiol, 2-methyl-3-furanthiol, and 2-sulfanylethyl acetate that contribute smoky, roasted meat, or coffee aromas to wines have also been isolated, but the sources of these compounds remain unclear^178,186,187^.

Unlike the majority of volatile compounds important for grape aroma, 3MH, 3MHA, 4MMP, and 4MMPOH do not exist in volatile form nor as glycosylated conjugates in the berry. Rather, the metabolic action of yeast is necessary to convert the precursors to the volatile compounds^20,188^. 3MHA is formed during fermentation through the esterification of 3MH by yeast enzymes and will not be discussed further^189^. The exact source of thiol precursors in wine remains disputed with several hypotheses involving cysteine and/or glutathione conjugates of grape origin. Alternatively or simultaneously, thiols may be generated through the addition of a sulfhydryl functional group from hydrogen sulfide to an aliphatic compound like (*E*)-2-hexenal^190^. For the latter hypothesis, the (*E*)-2-hexenal starting compound originates from the lipoxygenase pathway and its biosynthesis has been discussed in that section of this review.

Glutathione and cysteine conjugates of 3MH and 4MMP have been observed from grape juice. Both glutathione-conjugated and cysteine-conjugated precursors can undergo β-lyase-catalyzed cleavage by yeast to release the free polyfunctional thiol^157,180,191,192^. However, several studies have found limited correlation between juice concentrations of precursors and thiol levels in the resulting wine^193,194^. The addition of precursors does have a significant impact on final thiol concentrations but only a small amount of precursor is actually converted to a volatile form^188,192^. In addition, no correlation between the biosynthesis of 3MH and 4MMP has been found^188^. It is possible that there are several sources of volatile thiols in Sauvignon blanc wines. While the exact pathway responsible for the formation of volatile thiols remains unresolved, what limited genetic evidence exists supports some contribution from glutathione and cysteine precursors. Certainly, the occurrence of volatile thiols including 3MH in ripe guava (*Psidium guajava*) and yellow passionfruit (*Passiflora edulis* f. *flavicarpa*) implies the existence of a biosynthetic pathway for these molecules in plants^195,196^.

The proposed glutathione precursor of 3MH (3MH-glut) appears to result from conjugation of glutathione with (*E*)-2-hexenal through the action of a glutathione *S*-transferase (GST, Fig. 5b). GST expression can be induced by the presence of (*E*)-2-hexenal and other aldehydes. This links production of thiols to the lipoxygenase pathway^157,197,198^. Increases in glutathione and cysteine precursors have been found to coincide with greater accumulation of glutathione and (*E*)-2-hexenal immediately before harvest^35,199,200^. In plants, GSTs are a highly expanded gene family with diverse functions often related to detoxification and abiotic and biotic stress responses^201,202^. GSH conjugation of C_6_ aldehydes may be a mechanism to minimize cellular damage caused by the electrophilic nature of the compounds^203^.

Kobayashi et al. found that following exposure to UV-C radiation or downy mildew (*Plasmopara viticola*) infection, 3MH-glut accumulated in grape leaves and berries in correlation with the upregulation of *VviGST1, VviGST3*, and *VviGST4*^157^. Recombinant VviGST3 and VviGST4 catalyzed the formation of 3MH-glut from glutathione and (*E*)-2-hexenal, indicating that the two genes may be responsible for thiol precursor production in grapes^157^. However, more studies are necessary to determine if cultivar variations in *VviGST* sequences or expression are responsible for differences in thiol concentration. Interestingly, infection with *Botrytis cinerea* dramatically increases 4MMP and 3MH levels in Semillon^179,198^. *Botrytis* infection has been shown to cause upregulation of the lipoxygenase (LOX) pathway genes *VviLOXC* and *VviLOXO*, as well as 50 GST genes, including *VviGST3* and *VviGST4* in Semillon berries^152,204^. This supports the hypothesis that lipoxygenase pathway products are the precursors for glutathione conjugates of polyfunctional thiols.

The combined actions of two enzymes, a γ-glutamyltranspeptidase (GGT) and a carboxypeptidase, may convert glutathione conjugated thiols to their cysteine conjugates. Dubourdieu and Tominaga proposed that GGT catalyzes the removal of glutamate from 3MH-glut, resulting in the production of *S*-3-mercaptohexanol-L-cysteinylglycine (3MH-cysgly). This is followed by carboxypeptidase-catalyzed removal of the glycine to yield cysteine-conjugated 3MH (3MH-cys)^178^. Alternatively, a carboxypeptidase can react with 3MH-glut first, yielding a glutamylcysteinylated conjugate (3MH-glucys). This can then react with GGT and produce 3MH-cys (Fig. 5b). 3MH-cysgly has been shown to occur in grape juice, and the existence of 3MH-glucys was inferred by the production of 3MH-cys following the passage of Sauvignon blanc juice through a column containing immobilized GGT^191,205^.

3MH-glucys and 3MH-cysgly have also been found in the juice of yellow passionfruit, a fruit with high concentrations of 3MH^196^. Although no genes encoding a GGT or carboxypeptidase have been functionally characterized in grape, similar pathways in other organisms and the existence of the appropriate intermediates in grape suggest that they may be involved in the production of thiol precursors^157,178,180,206^. GGT-mediated catabolism of glutathione conjugates is well-documented in mammals and has also been shown to occur in plants; however, glutathione catabolism in plants is complicated and incompletely elucidated. GGTs are encoded by multiple genes in *Arabidopsis*, barley (*Hordeum vulgare*), and maize (*Zea mays*)^206^. In *Arabidopsis*, knockout mutants of *GGT3* (At4g29210) are unable to metabolize GSH conjugates in the roots^207^. Kobayashi et al. measured an increase in *VviGGT* expression in grape leaves following UV-C exposure but were unable to express a recombinant protein and test its catalytic activity^157^. Blanco-Ulate et al. found that two putative carboxypeptidases and a *GGT* are upregulated during *Botrytis* infection^204^. More research is necessary to determine which genes are responsible for thiol precursor biosynthesis and how their activity and expression differs between cultivars.

Even though very little is known about the genes involved in production of thiol precursors, many of the cultivars known for thiol content share parent-offspring or sibling relationships^5,56,178,208^. The shared ancestry between many thiol-rich cultivars suggests that there may be common alleles responsible for increased production of thiol precursors. Alternatively, the potential for thiol formation may be present in a number of unrelated varieties and has gone unnoticed due to their low concentration.

## Phenylpropanoids

The phenylpropanoid pathway is well known in grapes as the starting point for the biosynthesis of flavonoids, anthocyanins, stilbenes, hydroxycinnamates, and other important compounds^209,210^. Less well studied is the biosynthesis of volatile phenylpropanoids despite their abundance in the glycosidically bound fraction of volatile compounds in grape juice^11^. As with lipoxygenase pathway compounds, grape monoterpenol glycosyltransferases have been shown to generate glucosides with phenylpropanoid compounds as substrates; however, it is not known how much they contribute to the pool of bound phenylpropanoids in the berry^72^. As a group, phenylpropanoid-derived volatile compounds comprise up to 20% of volatile aglycones in enzymatically treated Chardonnay juice, albeit individual compounds generally do not exceed detection threshold. They may still contribute to the overall sensory properties of a wine since some, such as phenylethanol and eugenol, are also produced by microbes during fermentation and aging^11,211^. Although the mechanisms for their formation in grapevine have not been well studied, the phenylpropanoid volatiles found in grape are not unique to the genus, and it is likely that the biosynthetic pathways are similar^21^.

Eugenol is a characteristic volatile component of cloves and is also widespread in many plant species including grape^11,212^. In basil (*Ocimum basilicum*), eugenol is synthesized from coniferyl acetate and NADPH^28^. Although present in *V. vinifera* berries, eugenol is more abundant in non-*vinifera* species^3^. While a eugenol synthase gene has not been isolated and characterized in grape, transcript levels of an enzyme identified from a *M. rotundifolia* proteome as eugenol synthase were found to increase during ripening^213^. However, this was not accompanied by data regarding eugenol concentrations.

Of the phenylpropanoid-derived aroma compound in grapes, the biosynthesis of methyl anthranilate is best studied. Methyl anthranilate is an important component of the odor of *V. labrusca* cultivars and contributes to the “foxy” character of *V. labrusca* cultivars and hybrids despite its relatively low concentration compared with other esters^3,8^. Although primarily associated with American *Vitis* species and their hybrids, methyl anthranilate has also been detected from Pinot noir wine along with ethyl esters of anthranilic and cinnamic acids^214^. In *V. labrusca*, methyl anthranilate is synthesized from anthraniloyl-coA and methanol by an anthraniloyl-coenzyme A:methanol acyltransferase (*AMAT*). *AMAT* expression begins at véraison and peaks 16 weeks after flowering in the berry mesocarp, paralleling the enzyme activity, as well as the accumulation of methyl anthranilate and its precursor anthranilic acid^8^.

Anthranilic acid is produced by anthranilate synthase (AS) in the first step of tryptophan biosynthesis from chorismate^215^. Tryptophan concentration increases during ripening along with the concentration of anthranilic acid; however, it is unknown whether the increase in anthranilic acid is due to higher AS activity or tryptophan metabolism^8,216^. To produce methyl anthranilate, anthranilic acid is esterified with methanol supplied by methylesterase-mediated hydrolysis of pectins. Although other *V. labrusca* cultivars have similar *AMAT* expression and enzymatic activity to Concord, methyl anthranilate concentration appears to be limited by the rate of formation of the anthraniloyl-coA and methanol precursors^8^. Concord had the highest methanol content and pectin-methylesterase activity among five *V. labrusca* cultivars and hybrids studied^217^. The segregation ratio of methyl anthranilate concentrations in two families of crosses between cultivars with high and low methyl anthranilate content suggests that three dominant complementary genes are responsible^177^. Tryptophan metabolism is also important in the production of 2’-aminoacetophenone (2-AAP), another compound responsible for the “foxy” aroma of *V. labrusca* and *M. rotundifolia*.

The presence of methyl anthranilate, 2-AAP, and high total ester content in *V. labrusca* and *M. rotundifolia* and their relative absence in other grape species may be an adaptation for mammalian seed dispersal. Methyl anthranilate and 2-AAP act as bird repellents and olfactory attractants to mammals^218,219^. 2-AAP has also been implicated as the cause of “untypical aging off-flavor” in wines made from *V. vinifera*. Described as having an odor of “wet wool”, “furniture polish”, or “mothballs”, 2-AAP in wine is thought to be the result of oxidative degradation of indole-3-acetic acid (IAA), an important tryptophan-derived auxin^216,220^. Despite its presence in wine, 2-AAP has not been reported from *V. vinifera* berries and the biosynthetic pathway in *V. labrusca* and *M. rotundifolia* has not been explored to date.

## Conclusions

Although there are still many unanswered questions regarding the biosynthesis of aroma compounds, our understanding of the genes involved is steadily improving. A number of genes related to secondary metabolism have been functionally characterized in the years since Dunlevy et al.’s excellent 2009 review on aroma biosynthesis in grapes^21^. Knowledge of the genes involved in the biosynthesis of a particular aroma compound can help breeders select for desirable genotypes and raises the possibility of modulating the expression of these genes to attain a desired volatile profile in the resulting wine.

While the publication of the PN40024 reference genome has been invaluable in the identification of grapevine genes, it is not without its limitations. Reliance on a single genome sequence of a genotype for which we have no volatile compound data can be misleading due to structural variants that result in altered gene expression and function in other genotypes^85,221^. Drastic changes in aroma profile due to small mutations in key genes such as *VviDXS* or the VviGuaS variant of *VviTPS24* that are absent in PN40024 exemplify the limitations of a single reference genome^43,84,222^. Moreover, we cannot assume that a *V. vinifera* genome sequence is adequate for non*-vinifera* cultivars and hybrids. The recent publication of additional high-quality *V. vinifera* genomes will undoubtedly facilitate the discovery and characterization of genes and alleles responsible for varietal characteristics^221,223–225^.

Unlike many domesticated annual crops, *V. vinifera* cultivars have retained much of the diversity of wild grapevines, and the majority of cultivars are likely only a few generations removed from the wild progenitor^5,226,227^. Clonal propagation of selected grape cultivars has allowed for the spread of desirable genotypes; however this has also limited the distribution of potentially valuable rare mutations such as the REN1 powdery mildew (*Erysiphe necator*) resistance allele found in a few *V. vinifera* cultivars^4,228^. Over half of the accessions in the USDA germplasm collection are connected by first-degree relationships^5^. Worldwide, viticulture is characterized by the increased plantings of a small number of elite cultivars, resulting in limited exploitation of the genetic diversity available^9^. Not only does this underutilization of genetic resources result in vineyards susceptible to attack by insects and disease, genes for novel flavor compounds could exist in rare cultivars and wild species^5^. An understanding of the genetic bases for aroma can facilitate the application of *Vitis* genetic diversity towards developing a sustainable and dynamic grape and wine industry.
